# Joint international consensus statement for ending stigma of obesity

**DOI:** 10.1038/s41591-020-0803-x

**Published:** 2020-03-04

**Authors:** Francesco Rubino, Rebecca M. Puhl, David E. Cummings, Robert H. Eckel, Donna H. Ryan, Jeffrey I. Mechanick, Joe Nadglowski, Ximena Ramos Salas, Phillip R. Schauer, Douglas Twenefour, Caroline M. Apovian, Louis J. Aronne, Rachel L. Batterham, Hans-Rudolph Berthoud, Camilo Boza, Luca Busetto, Dror Dicker, Mary De Groot, Daniel Eisenberg, Stuart W. Flint, Terry T. Huang, Lee M. Kaplan, John P. Kirwan, Judith Korner, Ted K. Kyle, Blandine Laferrère, Carel W. le Roux, LaShawn McIver, Geltrude Mingrone, Patricia Nece, Tirissa J. Reid, Ann M. Rogers, Michael Rosenbaum, Randy J. Seeley, Antonio J. Torres, John B. Dixon

**Affiliations:** 1 King’s College London, Department of Diabetes, School of Life Course Science, London, UK; 2 King’s College Hospital, Bariatric and Metabolic Surgery, London, UK; 3 Rudd Center for Food Policy & Obesity, University of Connecticut, Hartford, CT USA; 4 UW Medicine Diabetes Institute, University of Washington, Seattle, WA USA; 5 Weight Management Program, Virginia Puget Sound Health Care System, University of Washington, Seattle, WA USA; 6 Division of Endocrinology, Metabolism & Diabetes, University of Colorado Anschutz Medical Campus, Aurora, CO USA; 7 Division of Cardiology, University of Colorado Anschutz Medical Campus, Aurora, CO USA; 8 Pennington Biomedical Research Center, Louisiana State University, Baton Rouge, LA USA; 9 The Marie-Josee and Henry R. Kravis Center for Clinical Cardiovascular Health at Mount Sinai Heart, New York, NY USA; 10 Divisions of Cardiology and Endocrinology, Diabetes and Bone Disease, Icahn School of Medicine at Mount Sinai, New York, NY USA; 11 Obesity Action Coalition, Tampa, FL USA; 12 Obesity Canada, Edmonton, Canada; 13 European Association for the Study of Obesity, Teddington, UK; 14 Diabetes UK, London, UK; 15 Boston University School of Medicine, Boston, MA USA; 16 Center for Nutrition and Weight Management, Boston Medical Center, Boston, MA USA; 17 Comprehensive Weight Control Center, Division of Endocrinology, Diabetes and Metabolism, Weill Cornell Medicine, New York, NY USA; 18 National Institute of Health Research, University College London Hospitals Biomedical Research Centre, London, UK; 19 University College London Hospital Foundation Trust, London, UK; 20 Centre for Obesity Research, Department of Medicine, University College London, London, UK; 21 Neurobiology of Nutrition and Metabolism Department, Pennington Biomedical Research Centre, Louisiana State University System, Baton Rouge, LA USA; 22 Centro de Innovación Clinica Las Condes Universidad Adolfo Ibañez, Santiago, Chile; 23 Department of Internal Medicine, University of Padova, Padua, Italy; 24 Hasharon Hospital-Rabin Medical Center, Sackler School of Medicine, Tel-Aviv University, Tel-Aviv, Israel; 25 Obesity Management Task Force, European Association for the Study of Obesity, Teddington, UK; 26 Department of Medicine, Indiana University School of Medicine, Indianapolis, IN USA; 27 Department of Surgery, Stanford School of Medicine and Palo Alto Virginia Health Care System, Stanford, CA USA; 28 School of Psychology, University of Leeds, Leeds, UK; 29 Scaled Insights, Nexus, University of Leeds, Leeds, UK; 30 Department of Health Policy & Management, Center for Systems & Community Design, New York, NY USA; 31 NYU-CUNY Prevention Research Center, Graduate School of Public Health & Health Policy, City University of New York, New York, NY USA; 32 Obesity, Metabolism and Nutrition Institute, Massachusetts General Hospital, Boston, MA USA; 33 Integrated Physiology and Molecular Medicine, Pennington Biomedical Research Center, Baton Rouge, LA USA; 34 Department of Medicine, Columbia University Irving Medical Center, New York, NY USA; 35 ConscienHealth, Pittsburgh, PA USA; 36 Division of Endocrinology, Columbia University, New York, NY USA; 37 Diabetes Complications Research Centre, University College Dublin, Dublin, Ireland; 38 Government Affairs & Advocacy, American Diabetes Association, Arlington, VA USA; 39 Fondazione Policlinico Universitario A. Gemelli, IRCCS, Rome, Italy; 40 Catholic University, Rome, Italy; 41 Division of Endocrinology, Diabetes & Metabolism, Columbia University College of Physicians and Surgeons, New York, NY USA; 42 Pennsylvania State Hershey Medical Center, Hershey, PA USA; 43 Columbia University Irving Medical Center, New York, NY USA; 44 Department of Surgery, University of Michigan, Ann Arbor, MI USA; 45 Hospital Clinico San Carlos. Universidad Complutense de Madrid, Madrid, Spain; 46 Baker IDI Heart and Diabetes Institute, Melbourne, Australia

**Keywords:** Diabetes, Metabolic syndrome, Obesity

## Abstract

People with obesity commonly face a pervasive, resilient form of social stigma. They are often subject to discrimination in the workplace as well as in educational and healthcare settings. Research indicates that weight stigma can cause physical and psychological harm, and that affected individuals are less likely to receive adequate care. For these reasons, weight stigma damages health, undermines human and social rights, and is unacceptable in modern societies. To inform healthcare professionals, policymakers, and the public about this issue, a multidisciplinary group of international experts, including representatives of scientific organizations, reviewed available evidence on the causes and harms of weight stigma and, using a modified Delphi process, developed a joint consensus statement with recommendations to eliminate weight bias. Academic institutions, professional organizations, media, public-health authorities, and governments should encourage education about weight stigma to facilitate a new public narrative about obesity, coherent with modern scientific knowledge.

## Main

Individuals with obesity face not only increased risk of serious medical complications but also a pervasive, resilient form of social stigma. Often perceived (without evidence) as lazy, gluttonous, lacking will power and self-discipline, individuals with overweight or obesity are vulnerable to stigma and discrimination in the workplace, education, healthcare settings, and society in general. Weight stigma can cause considerable harm to affected individuals, including physical and psychological consequences. The damaging impact of weight stigma, however, extends beyond harm to individuals. The prevailing view that obesity is a choice and that it can be entirely reversed by voluntary decisions to eat less and exercise more can exert negative influences on public health policies, access to treatments, and research^1–3^.

Although raising awareness of the negative consequences of weight stigma is important, awareness alone is not sufficient to eliminate the issue. Challenging and changing widespread, deep-rooted beliefs, longstanding preconceptions, and prevailing mindsets requires a new public narrative of obesity that is coherent with modern scientific knowledge. Given the pervasiveness of societal weight bias, this goal can only be achieved through the concerted efforts of a broad group of stakeholders, including healthcare providers (HCPs), researchers, the media, policymakers, and patients.

To best inform HCPs, policymakers, and the public about stigma associated with obesity, a multi-disciplinary group of international experts, including representatives of ten scientific organizations (Table 1), reviewed available evidence on the causes and harms of weight stigma, developing a joint consensus statement with recommendations to eliminate weight bias.

**Table 1:** Composition of the expert panel

Name	Affiliation	Country
Caroline M. Apovian	Boston University School of Medicine	USA
Louis J. Aronne	Weill Cornell Medicine	USA
Rachel Batterham	University College London Hospitals	UK
Hans-Rudolf Berthoud	Pennington Biomedical Research Centre	USA
Camilo Boza	Universidad Adolfo Ibañez	Chile
Luca Busetto	University of Padova	Italy
David E. Cummings^a^	University of Washington	USA
Dror Dicker	Sackler School of Medicine	Israel
Mary de Groot	Indiana University School of Medicine	USA
John B. Dixon^a^	Baker Heart and Diabetes Institute	Australia
Robert H. Eckel	University of Colorado	USA
Dan Eisenberg	Stanford School of Medicine	USA
Stuart W. Flint	University of Leeds	UK
Terry T. Huang	City University of New York	USA
Lee M. Kaplan	Harvard Medical School	USA
John P. Kirwan	Pennington Biomedical Research Center	USA
Judith Korner	Columbia University	USA
Theodore K. Kyle	ConscienHealth	USA
Blandine Laferrère	Columbia University	USA
Carel W. le Roux	University College Dublin, Ireland	Ireland
LaShawn McIver	American Diabetes Association	USA
Mechanick I. Jeffrey	Mount Sinai Health System	USA
Geltrude Mingrone	Universita Cattolica Del Sacro Cuore	Italy
Joe Nadglowski	Obesity Action Coalition	USA
Patricia Nece	Obesity Action Coalition	USA
Rebecca M. Puhl	University of Connecticut	USA
Ximena Ramos Salas	Obesity Canada	Canada
Tirissa J. Reid	Columbia University	USA
Ann M. Rogers	Penn State Hershey Medical Center	USA
Michael Rosenbaum	Columbia University	USA
Rubino Francesco^a,b^	King’s College London	UK
Donna H. Ryan^a^	Pennington Biomedical Research Center	USA
Philip R. Schauer	Pennington Biomedical Research Center	USA
Randy J. Seeley	University of Michigan	USA
Antonio Torres	Universidad Complutense de Madrid	Spain
Douglas Twenefour	Diabetes UK	UK

^a^Co-chairs.

^b^Conference director–convenor.

Moderator: Olivia Barata Cavalcanti (non-voting).

A specific goal—representing novelty from previous related initiatives—was to address the gap between stigmatizing narratives around obesity and current scientific knowledge regarding mechanisms of body-weight regulation. The overarching objective was to gather a broad group of experts and scientific organizations to recognize the problem and, to our knowledge for the first time, ‘speak with one voice’ about this important issue.

Here we report the conclusions of this exercise and resulting joint consensus statements, with a call to action for all stakeholders to take a pledge (Box 1) to end weight stigma and discrimination.

Box 1 Pledge to eliminate weight bias and stigma of obesity **We recognize that**

- Individuals affected by overweight and obesity face a pervasive form of social stigma based on the typically unproven assumption that their body weight derives primarily from a lack self-discipline and personal responsibility.
- Such portrayal is inconsistent with current scientific evidence demonstrating that body-weight regulation is not entirely under volitional control, and that biological, genetic, and environmental factors critically contribute to obesity.
- Weight bias and stigma can result in discrimination, and undermine human rights, social rights, and the health of afflicted individuals.
- Weight stigma and discrimination cannot be tolerated in modern societies.

**We condemn**

- The use of stigmatizing language, images, attitudes, policies, and weight-based discrimination, wherever they occur.

**We pledge**

- To treat individuals with overweight and obesity with dignity and respect.
- To refrain from using stereotypical language, images, and narratives that unfairly and inaccurately depict individuals with overweight and obesity as lazy, gluttonous, and lacking willpower or self-discipline.
- To encourage and support educational initiatives aimed at eradicating weight bias through dissemination of current knowledge of obesity and body-weight regulation.
- To encourage and support initiatives aimed at preventing weight discrimination in the workplace, education, and healthcare settings.

## Methodology

### Partner organizations and selection of voting delegates

The consensus-development conference was convened by F.R. (conference director), jointly with the following partner organizations: American Association of Clinical Endocrinologists, American Association for Metabolic and Bariatric Surgery, American Diabetes Association, Diabetes UK, European Association for the Study of Obesity, International Federation for the Surgery of Obesity and Metabolic Disorders, Obesity Action Coalition, Obesity Canada, The Obesity Society, and World Obesity Federation.

The conference director (F.R.), co-chairs (D.E.C., D.H.R., and J.B.D.), and the partner organizations appointed a multidisciplinary expert panel (Table 1) of 36 internationally recognized academics representing multiple scientific disciplines, including endocrinology, nutrition, internal medicine, surgery, psychology, molecular biology, cardiology, gastroenterology, primary care, public health, and health policy. The expert panel also included patient-advocacy experts, plus an individual with obesity to speak on behalf of patients. Given that the main aim of this consensus conference was not to define weight stigma or assess it from an ethical perspective, we did not include ethicists in the expert panel.

The 36 members of the expert panel, who constituted voting delegates for the entire consensus-development process, were selected primarily from academics with documented expertise about the topics of the conference and relevant publication records. Each of the ten partner societies used their own criteria to choose their representatives for the expert panel. Criteria included, expertise in weight stigma, obesity, and/or previous participation in relevant committees or initiatives related to weight stigma. One independent, non-voting moderator (O.B.C.) with previous experience in Delphi methodology administered questionnaires for the modified Delphi process and chaired the face-to-face meeting of voting delegates.

### Review of evidence

A subgroup of expert panel members (F.R., D.E.C., and J.B.D.) contributed to a review of scientific publications in Medline on a broad set of topics related to weight bias, stigma, and discrimination. Specific, preset research questions for the review of evidence included: (i) prevalence of weight bias and stigma (in the media, healthcare, education, and workplace); (ii) psychologically and physically harmful effects on individuals; (iii) impact on access to care and research, and evidence of workplace weight discrimination; (iv) biological mechanisms of weight regulation in physiology and disease; (v) clinical evidence of uptake and barriers to access of available treatments; and (vi) mechanisms of body-weight regulation and energy homeostasis. We also searched broader data sources for references to weight discrimination in current legislation.

Members of the expert panel with specific expertise (R.M.P., C.A., L.J.A., D.H.R., T.J.R., and S.W.F.) were also tasked to independently conduct a short narrative review of current knowledge on one of the above research questions, according to their specific expertise.

Given the objectives of the consensus conference, the diversity of the subjects under consideration, and the variance in quality of evidence across disciplines, we considered a broad evidence base, including previously published systematic reviews, and various types of observational studies, experimental medicine, and translational studies in animals (weight-regulation mechanisms). For evidence about media portrayal of obesity, we used a 2010 review based on PsycINFO database searches using weight bias and stigmatization-related terms and phrases to identify journal articles published in English between 1994 and 2009 (ref. ^4^). We also identified papers related to these subjects in Medline published between 2011 and 2019. To assess beliefs about obesity, type 2 diabetes, and related treatment options, we used research surveys and pools of opinion conducted among HCPs and/or the general public, and reported in Medline.

A document with the results of this review was circulated among the rest of the group in preparation for the modified Delphi process, seeking further input.

### Delphi-like consensus-development process

Based on results of the review of evidence, a subgroup of expert panel members with special expertise in specific subtopics developed questionnaires, including a set of statements and recommendations that were believed to summarize and reflect available evidence. These questionnaires were then circulated among the expert panel. An online Delphi-like method was used to measure the degree of consensus for the statements and recommendations by a web-based survey tool (SurveyMonkey; https://www.surveymonkey.co.uk). We adapted the original Delphi method^5^ to the scopes and nature of this exercise. Unlike other Delphi studies, in which the first round consists mainly of open-ended questions, we used agree/disagree questions designed by a subgroup of members of the expert panel (F.R., S.W.F., D.E.C., and J.B.D.) for the first round.

Approximately three weeks before the survey was first administered, we informed potential participants of the objectives of the study, provided information about the Delphi process, and invited them to contribute. Participants were assured that responses were confidential, with individual responses known only to the impartial, non-voting survey moderator.

The moderator administered the questionnaires through two rounds of Delphi-like process. Delegates who did not agree with the proposed statements were asked to state their reasons and propose amendments. A further (third) round of Delphi was administered after the in-person meeting. All questions also contained a box for individual, non-compulsory comments.

Each survey round was conducted over two weeks: one week for response acquisition (including e-mail reminders before the closing date), and one week for data analysis and preparation of the subsequent round. A personalized email message was sent to each respondent with a URL link to the survey.

Response rate for both of the first two Delphi rounds was 34/36 expert panel members, and all 36 members responded in the third Delphi round.

### In-person consensus meeting

Supporting evidence and draft conclusions generated through the Delphi process were presented at the 4th World Congress on Interventional Therapies for Type 2 Diabetes 2019 (WCITD2019, New York, 10 April, 2019). Proceedings were open to public comment by other experts in the field (WCITD2019 faculty members) and by the entire audience through opinion polls, using real-time electronic voting (Turning Technology software). On 10 April 10, 2019, voting delegates met face-to-face to review, amend, and vote on each consensus statement.

### Final steps

After the meeting in New York, the document with conclusions reached by the experts underwent a final review and approval through a third round of Delphi process.

The final consensus document, including the ‘Pledge to Eliminate Weight Bias and Stigma of Obesity’ (Box 1), was then submitted to the scientific committees and executive boards of partner organizations and other stakeholders for review and endorsement. A subgroup of the expert panel (F.R., R.M.P., D.E.C., R.H.E., D.H.R., J.I.M., J.N., X.R.S., P.R.S., D.T., and J.B.D.) generated the first draft of the report. The draft report was then circulated among all other members of the expert panel for further input and approval before submission for publication.

All members of the expert panel, all partner organizations, and additional organizations listed in Box 2 have formally endorsed the statement and taken the pledge to eliminate weight stigma.

Box 2 Organizations that have endorsed the statement and/or accepted to take the pledge to eliminate weight stigma as of 26 February 2020 **Partner organizations**

- American Association of Clinical Endocrinologists (AACE)
- American Association for Metabolic and Bariatric Surgery (ASMBS)
- American Diabetes Association (ADA)
- Diabetes UK
- European Association for the Study of Obesity (EASO)
- International Federation for the Surgery of Obesity and metabolic Disorders (IFSO)
- Obesity Action Coalition (OAC)
- Obesity Canada
- The Obesity Society (TOS), USA
- World Obesity Federation (WOF)

**Other scientific and patient societies**

- American Academy of Sleep Medicine (AASM)
- American Society for Nutrition (ASN)
- Association of British Clinical Diabetologists (ABCD)
- The Australian National Association of Clinical Obesity Services (NACOS)
- Australian and New Zealand Metabolic and Obesity Surgery Society (ANZMOSS)
- Austrian Society for Obesity and Metabolic Surgery
- Brazilian Society for Bariatric and Metabolic Surgery (SBCBM)
- British Obesity and Metabolic Surgery Society (BOMSS)
- Canadian Association of Cardiovascular Prevention and Rehabilitation (CACPR)
- Canadian Association of Occupational Therapists (Association Canadienne des ergothérapeutes)
- The Canadian Society of Endocrinology and Metabolism (CSEM)
- CIHR-SPOR Chair in Innovative, Patient-Oriented, Behavioural Clinical Trials
- Chilean Society for Bariatric and Metabolic Surgery
- Colegio Mexicano de Cirurgia Para la Obesidad y Enfermedades Metabolicas
- Croatian Society of Obesity
- Dietitians of Canada
- Dutch Society for Metabolic and Bariatric Surgery (DSMBS)
- The Endocrine Society (USA)
- European Coalition for People Living with Obesity (ECPO)
- French Clinical Research Network in Obesity (FORCE)
- French Society for Research and Care of Obesity (AFERO)
- French Society of Bariatric and Metabolic Surgery (SOFFCO-MM)
- Hellenic Medical Association for Obesity (HMAO)
- Hellenic Society for Bariatric and Metabolic Surgery
- Hellenic Society for the Study of Obesity, Metabolism and Eating Disorders
- Hong Kong Association for the Study of Obesity
- Hong Kong Obesity Society
- Hong Kong Society for Metabolic and Bariatric Surgery
- Hungarian Society for the Study of Obesity
- International Behavioural Trials Network (IBTN)
- International Society for the Perioperative Care of the Obese Patient (ISPCOP)
- Irish Society for Clinical Nutrition and Metabolism (IrSPEN)
- The Israeli Association for the Study of Obesity
- Italian Obesity Society (SIO)
- Korean Society for the Study of Obesity (KSSO)
- Latin American Federation of Obesity (FLASO)
- The Lithuanian Society of Bariatric Surgery
- Mexican Society of Obesity
- National Lipid Association (USA)
- Norwegian Society for the Surgery of Obesity
- Obesity Australia
- Obesity Care Advocacy Network (OCAN)
- Obesity Collective
- Obesity Medicine Association (USA)
- Obesity Society of Nigeria
- The Obesity Surgery Society India (OSSI)
- Obesity UK
- Romanian Federation of Diabetes, Nutrition, Metabolic diseases
- The Royal College of Physicians -RCP- (UK)
- Russian Society of Bariatric Surgeons
- Sociedad Argentina de Cirugia de la Obesidad Enfermedad Metabolica y Otras Relacionados con la Obesidad
- Sociedad Argentina de Obesidad y Trastornos Alimentarios
- Sociedad Española de Cirugía de la Obesidad y Enfermedades Metabólicas (SECO)
- Society of American Gastrointestinal and Endoscopic Surgeons (SAGES)
- Society of Behavioral Medicine (SBM)
- The Society for Surgery of the Alimentary Tract (SSAT)
- South African Society for Surgery Obesity and Metabolism
- UK Association for the Study of Obesity

**Scientific and medical journals**

- *The Annals of Surgery*
- *Cell Metabolism* (Cell Press)
- *Cell Reports Medicine* (Cell Press)
- *Clinical Obesity*
- *The Lancet Diabetes & Endocrinology*
- *Med* (Cell Press)
- Nature Research (all journals)
- *Obesity*
- *Obesity Reviews*
- *Obesity Science and Practice*
- *Obesity Surgery*
- *Pediatric Obesity*
- *Surgery for Obesity and Related Diseases* (SOARD)
- *Trends in Endocrinology and Metabolism* (Cell Press)
- *Trends in Molecular Medicine* (Cell Press)

**Academic institutions and hospitals**

- Baker Heart and Diabetes Institute. Melbourne, Australia
- The Charles Perkins Institute, University of Sydney (Australia)
- Geisinger Obesity Institute, Geisinger Health System, Danville, PA (USA)
- King’s College Hospital NHS Foundation Trust (UK)
- King’s College London (UK)
- London Bridge Hospital, London (UK)
- Pennington Biomedical Research Center (USA)
- Specialized Centers for Obesity Management (GCC-CSO) (France)
- St Vincent Private Hospital, Dublin (Ireland)
- Summer M. Redstone Center, Milken Institute School of Public Health, George Washington University (USA)
- Technische Universität Dresden; Faculty of Medicine Carl Gustav Carus (Germany)
- Transcampus of Technische Universität Dresden; Faculty of Medicine Carl Gustav Carus (Germany) and King’s College London
- University College London Hospitals NHS Foundation Trust (UK)
- University of Connecticut Rudd Center for Food Policy & Obesity (USA)
- The Veneto Obesity Network (Italy)

**Parliamentary groups**

- The All-Party Parliamentary Group on Obesity (APPG): a group of cross-party members of the House of Commons and House of Lords campaigning for improved prevention and treatment of obesity (UK)

### Descriptors of grade of consensus

A supermajority rule was used to define consensus. Consensus was considered to have been reached when > 67% of the experts agreed on a given topic. However, language was iteratively modified to maximize agreement, and the degree of consensus for each statement was graded according to the following scale: grade U was 100% agreement (unanimous); grade A was 90–99% agreement; grade B was 78–89% agreement; and grade C was 67–77% agreement. This grading scale is meant to indicate statements that reflect unanimous or near-unanimous opinions (grades U and A), strong agreement with little variance (grade B), or a consensus statement that reflects an averaging of more and possibly extremely diverse opinions (grade C). All statements included in this consensus document achieved either grades U or A, which we report for each statement.

The first questionnaire asked 58 questions, including six on expert panel demographic information. During the three Delphi-like rounds and the in-person voting session, the expert panel eliminated five consensus questions that were deemed to be duplicative or redundant. Our iterative changes throughout the process yielded 47 final statements (Tables 2 and 3), all with > 89% consensus (grades A and U), as summarized in Box 3.

**Table 2:** Consensus statements on the stigma of obesity

Item	Topic	Grade
**1. Prevalence of weight stigma and weight-based discrimination**		
1. 1	A substantial body of evidence demonstrates that weight-based stigma is extremely pervasive among people of diverse ages and backgrounds.	A
1.2.	People with obesity are often subject to unfair treatment and discrimination in the workplace, education, and healthcare settings.	U
1.3.	Weight-based discrimination is one of the most common forms of discrimination in modern societies.	A
1.4.	Women are more likely to suffer weight-based discrimination compared to men; this has the potential to contribute to inequalities in employment and education.	A
**2. Weight stigma and the media**		
2.1	Media portrayal of obesity is influential; it plays an important role in shaping public attitudes and beliefs about people with obesity and related diseases.	U
2.2	In news media, obesity is frequently attributed to personal responsibility, and afflicted individuals are often represented—without evidence—as being lazy, gluttonous, and lacking will power and self-discipline.	U
2.3	Media portrayals of diet and exercise as the only appropriate therapies for obesity are scientifically inaccurate and may deter patients from pursuing additional evidence-based interventions.	U
2.4	Media portrayals that encourage weight-based stigma and discrimination are harmful and should be discouraged.	U
**3. Weight stigma in healthcare**		
3.1	Many healthcare professionals hold negative attitudes about obesity, including stereotypes that affected patients are lazy, lack self-control and willpower, are personally to blame for their weight, and are noncompliant with treatment.	A
3.2	Weight-based stigma among healthcare professional is unacceptable, especially among those who are specialized in the care of people with obesity.	A
3.3	Many healthcare facilities are inadequately equipped to treat patients with obesity.	U
**4. Weight-based discrimination**		
4.1	Obesity stigmatization and discrimination occur in schools, such that children and adolescents living with obesity are at an increased risk for poor peer relations and experience high rates of bullying.	A
4.2	Weight-based stigma unfairly undermines opportunities for employment, career progression, and income for people with obesity.	A
4.3	For most people with obesity who experience discrimination in recruitment or in the workplace, there is no legal protection under current legislation.	A
**5. Physical and mental health consequences**		
5.1	Weight-based stigma and internalized weight bias can be particularly harmful to mental health, increasing risks of depressive symptoms, anxiety, and promoting lower self-esteem, social isolation, stress, and substance use.	U
5.2	Adults and children who experience weight-based stigma are more likely to avoid exercise and physical activity, and to engage in unhealthy diets and sedentary behaviors that increase the risk of worsening obesity.	A
**6. Quality of care, access to care**		
6.1.	Quality of health care is adversely affected by weight-based stigma.	U
6.2	Fear of prejudice and internalized weight bias cause direct and indirect harm to patients with obesity, as they are less likely to seek and receive appropriate treatment for obesity or other conditions.	A
6.3.	Despite the well-recognized risks of obesity and related illnesses, it is common for health insurance companies to have significant limitations or complete lack of coverage for evidence-based treatments of obesity—especially metabolic surgery. These policies can cause harm, are indefensible, and are ethically objectionable.	A
**7. Weight stigma and public health**		
7.1	Despite significant evidence that weight-based stigma causes damage to individuals and society, public health efforts to date have not widely addressed stigma as a barrier to combat the obesity epidemic.	A
7.2	Stigmatizing public health campaigns that emphasize the role of personal responsibility and ‘healthy lifestyle’ choices focusing only on nutrition and physical activity overlook important societal and environmental factors that critically contribute to the epidemic of obesity.	A
7.3	Some public health campaigns appear to embrace stigmatization of individuals with obesity as a means to motivate behavior change and achieve weight loss through self-directed diet and increased physical exercise. These approaches are not supported by scientific evidence, and they risk further increasing societal discrimination against people with obesity, yielding the opposite to the intended effect.	A
**8. Weight stigma and research**		
8.1	Misconceptions about the causes of obesity are likely to play an important part in public support for obesity research and relative allocation of public funding, compared to other diseases that are believed not to depend on factors completely controllable by individuals’ actions (for example, cancer, infectious diseases, etc.).	A
8.2.	Diseases such as obesity and type 2 diabetes receive far less research funding than do other chronic diseases, relative to their prevalence and the costs they impose upon society.	A
**9. Causes and contributors of weight stigma/discrimination**		
9.1	Causal attributions of personal responsibility for obesity are associated with stronger weight bias, whereas lower levels of weight-based stigma are associated with stronger beliefs in genetic/physiological or environmental causes of obesity.	U
9.2	The absence of policies to prohibit weight discrimination communicates a message that weight stigma is acceptable and tolerable, thus reinforcing weight-based inequities.	A
9.3	The idea that the causes of obesity depend on individuals’ faults, such as laziness and gluttony, provides the foundation for stigma against obesity.	A
**10. The science of obesity versus misconceptions in the public narrative of obesity**		
10.1	The assumption that body weight is entirely under volitional control, and that voluntarily eating less and/or exercising more can entirely prevent or reverse obesity is at odds with a definitive body of biological and clinical evidence developed over the last several decades.	U
10.2	Popular expressions such as ‘energy in versus energy out’ or ‘calories in versus calories out’ are misleading because they inaccurately imply that body weight and/or fat mass are solely influenced by the number of food calories ingested, and the amount of energy burned through exercise. This narrative is not supported by evidence and provides a foundation for popular, stigmatizing views that blame individuals’ lack of willpower for their obesity.	A
10.3	The idea that obesity is a ‘choice’ is a misconception, inconsistent with both logic and scientific evidence showing that obesity results primarily from a combination of genetic, epigenetic, and environmental factors.	A
10.4	There is a widespread assumption, including among many medical professionals, that voluntary lifestyle changes (diet and exercise) can entirely reverse obesity over long periods of time, even when severe. This assumption runs contrary to indisputable scientific evidence demonstrating that voluntary efforts to reduce body weight activate potent compensatory biologic responses (for example, increased appetite, decreased metabolic rate) that typically promote long-term weight regain.	A
10.5	Metabolic surgery is not an ‘easy way out’ but an evidence-based, physiologic approach to treating obesity and type 2 diabetes, given its ability to influence underlying mechanisms of energy and glucose homeostasis.	A
**11. Obesity: ‘condition’ or ‘disease’?**		
11.1	There is objective evidence that in many patients, obesity presents the typical attributions of a disease status, which include specific signs and/or symptoms, distinct pathophysiology, reduced quality of life, and increased risk of complications/mortality.	U
11.2	Although prevailing evidence supports a rationale for obesity to be defined as a disease, as recognized by leading worldwide authority bodies and medical associations, current diagnostic criteria for obesity (only based on BMI levels) are inadequate to accurately diagnose obesity.	A

**Table 3:** Consensus statements on the stigma of obesity: recommendations

Item	Topic	Grade
**Generalities**		
1	Weight-based stigma and obesity discrimination should not be tolerated in education, healthcare, or public-policy sectors.	U
2	Explaining the gap between scientific evidence and the conventional narrative of obesity built around unproven assumptions and misconceptions may help reduce weight bias and alleviate its numerous harmful effects.	A
3	The conventional narrative of obesity built around unproven assumptions of personal responsibility, and misconceptions about the causes and remedies of obesity causes harm to individuals and to society. Media, policy makers, educators, HCPs, academic Institutions, public health agencies, and government must ensure that the messages and narrative of obesity are free from stigma and coherent with modern scientific evidence.	A
4	Obesity should be recognized and treated as a chronic disease in healthcare and policy sectors.	A
**Media**		
5	We call on the media to produce fair, accurate, and non-stigmatizing portrayals of obesity. A commitment from the media is needed to shift the narrative around obesity.	U
**Healthcare and education of HCPs**		
6	Academic institutions, professional bodies, and regulatory agencies must ensure that formal teaching on the causes, mechanisms, and treatments of obesity are incorporated into standard curricula for medical trainees, and other HCPs.	U
7	HCPs specialized in treating obesity should provide evidence of stigma-free practice skills. Professional bodies should encourage, facilitate, and develop methods to certify knowledge of stigma and its effects, along with stigma-free skills and practices.	A
8	Given the prevalence of obesity and obesity-related diseases, appropriate infrastructure for the care and management of people with obesity, including severe obesity, must be standard requirement for accreditation of medical facilities and hospitals.	U
**Public health**		
9	Public health practices and messages should not use stigmatizing approaches to promote anti-obesity campaigns. These practices are objectively harmful and should be banned.	A
10	Public health authorities should identify and reverse policies that promote weight-based stigma, while increasing scientific rigor in obesity-related public policy.	A
**Research**		
11	Research in obesity and type 2 diabetes should receive appropriate public funding, commensurate to their prevalence and impact on human health and society.	A
**Policies and legislation**		
12	There should be strong and clear policies to prohibit weight-based discrimination.	U
13	Policies and legislation to prohibit weight discrimination are an important and timely priority to reduce or eliminate weight-based inequities.	U

In this document, we use the terms ‘weight stigma’, ‘weight-based stereotypes’, or ‘weight bias’ to refer to biases against individuals with overweight and obesity, not underweight. We provide definitions in Box 4.

Box 3 Executive summary (Grade of consensus (GoC): U is unanimous; A is >90% consensus)

Weight stigma is reinforced by misconceived ideas about body-weight regulation and lack of awareness of current scientific evidence. Weight stigma is unacceptable in modern societies, as it undermines human rights, social rights, and the health of afflicted individuals (GoC: A).

Research indicates that weight stigma can cause significant harm to affected individuals. Individuals who experience it suffer from both physical and psychological consequences, and are less likely to seek and receive adequate care (GoC: U).

Despite scientific evidence to the contrary, the prevailing view in society is that obesity is a choice that can be reversed by voluntary decisions to eat less and exercise more. These assumptions mislead public health policies, confuse messages in popular media, undermine access to evidence-based treatments, and compromise advances in research (GoC: A).

For the reasons above, weight stigma represents a major obstacle in efforts to effectively prevent and treat obesity and type 2 diabetes. Tackling stigma is not only a matter of human rights and social justice, but also a way to advance prevention and treatment of these diseases (GoC: A).

Academic institutions, professional organizations, media, public health authorities, and government should encourage education about weight stigma and facilitate a new public narrative of obesity, coherent with modern scientific knowledge (GoC: U).

Box 4 Definitions **Weight stigma** refers to social devaluation and denigration of individuals because of their excess body weight, and can lead to negative attitudes, stereotypes, prejudice, and discrimination.

**Weight-based stereotypes** include generalizations that individuals with overweight or obesity are lazy, gluttonous, lacking in willpower and self-discipline, incompetent, unmotivated to improve their health, non-compliant with medical treatment, and are personally to blame for their higher body weight.

**Weight discrimination** refers to overt forms of weight-based prejudice and unfair treatment (biased behaviors) toward individuals with overweight or obesity.

**Weight bias internalization** occurs when individuals engage in self-blame and self-directed weight stigma because of their weight. Internalization includes agreement with stereotypes and application of these stereotypes to oneself and self-devaluation^6^.

**Explicit weight bias** refers to overt, consciously held negative attitudes that can be measured by self-report.

**Implicit weight bias** consists of automatic, negative attributions and stereotypes existing outside of conscious awareness.

## Summary of evidence

### Prevalence of weight bias, stigma, and discrimination

Substantial research has demonstrated that weight stigma and discrimination are pervasive, global issues^7,8^. Weight stigma has been documented in multiple societal domains, including the workplace, education, healthcare settings, and within families^9,10^. Stigma has persisted despite the markedly increased prevalence of obesity in recent decades. Among adults with obesity, the prevalence of weight discrimination is 19–42%, with higher rates among those with higher body-mass index (BMI), and among women compared with men^11–13^.

Estimates from a 2018 study suggest that approximately 40–50% of US adults with overweight and obesity experience internalized weight bias, and about 20% of US adults experience this at high levels^14^. Internalized weight bias is present in individuals across diverse body-weight categories, but especially among individuals with higher BMI who are trying to lose weight^14^.

Evidence suggests that the media is a pervasive source of weight bias and can reinforce stigma through the use of inaccurate framing of obesity and inappropriate images, language, and terminology that attribute obesity entirely to personal responsibility^15^. It has been estimated that over two thirds of images accompanying US media reports of obesity contain weight stigma, and experimental studies show that viewing these types of images leads to increased weight bias^16^.

Weight bias has been reported among HCPs in the United States and around the world, including among primary care providers, endocrinologists, cardiologists, nurses, dietitians, mental health professionals, medical trainees, and professionals engaged in research and clinical management of obesity^17,18^.

### Physical and mental health consequences of weight stigma

Children with overweight and obesity are frequently subject to weight-based teasing and bullying at school. Compared with students of lower body weight, adolescents with overweight or obesity are significantly more likely to experience social isolation^19–21^ and are at increased risk for relational, verbal, cyber, and physical victimization^22^. They are also more susceptible to developing mental health disorders, especially anxiety and depression, in addition to obesity, type 2 diabetes, and cardiovascular disease in later life^23^.

Weight stigma, rather than obesity itself, may be particularly harmful to mental health and is associated with depressive symptoms, higher anxiety levels, lower self-esteem, social isolation, perceived stress, substance use^24–26^, unhealthy eating and weight-control behaviors, such as binge eating and emotional overeating^27^. Experimental studies also show, paradoxically, that exposing individuals to weight stigma can lead to increased food intake, regardless of BMI^3,28^. Correlative and randomized-controlled studies also show that experience of weight stigma is linked with lower levels of physical activity, higher exercise avoidance^29–31^, consumption of unhealthy diets, and increased sedentary behaviors^1–3^, as well as increased obesity and weight gain over time^32^, and increased risk of transitioning from overweight to obesity in both adults and adolescents^33–35^.

Individuals with overweight and obesity who experience weight discrimination show higher levels of circulating C-reactive protein^36^, cortisol^37^, long-term cardio-metabolic risk^38^, and increased mortality^39^ compared with those who do not experienced weight discrimination.

### Quality of care and health care utilization

Evidence suggests that physicians spend less time in appointments and provide less education about health to patients with obesity compared with thinner patients^17^, and patients who report having experienced weight bias in the healthcare setting have poor treatment outcomes^40^ and might be more likely to avoid future care^41^. Obesity also adversely impacts age-appropriate cancer screening, which can lead to delays in breast, gynecological, and colorectal cancer detection^42^.

A thematic analysis of 21 studies examined the perceptions of weight bias and its impact on engagement with primary health care services^43^. Negative influences on engagement with primary care were evaluated and ten themes were identified: contemptuous, patronizing, and disrespectful treatment, lack of training, ambivalence, attribution of all health issues to excess weight, assumptions about weight gain, barriers to health care utilization, expectation of differential health care treatment, low trust and poor communication, avoidance or delay of health services, and seeking medical advice from multiple HCPs.

The widespread, but unproven, assumption that body weight is entirely controllable by lifestyle choices and that self-directed efforts can reverse even severe forms of obesity or type 2 diabetes^44^ could explain the low level of public support for coverage of anti-obesity interventions beyond diet and exercise^45^, regardless of their evidence base. For example, many public and private health insurers either do not provide coverage or have substantive limitations in the coverage of metabolic surgery, including fulfilment of a number of criteria for which there is limited or no clinical evidence^46,47^. These attitudes are in stark contrast with coverage of treatment for other chronic diseases (for example, cancer, heart disease, and osteoarthritis) that are not conditional to similar restrictions, and for which use of similarly arbitrary coverage criteria would be socially indefensible and ethically objectionable.

### Stigmatization of surgical treatment for obesity

Metabolic surgery (also known as bariatric surgery) provides a compelling example of how weight stigma can also extend to treatments for obesity. Compared with individuals who lose weight using diet and exercise alone, those who lose weight through metabolic surgery can be at risk of stronger stigma because they are stereotyped as being lazy and being less responsible for their weight loss^48,49^. It is not surprising that many hide their surgical status^49^.

Despite evidence of efficacy and cost-effectiveness^50,51^ of surgical interventions for obesity, only 0.1–2% of surgical candidates who qualify worldwide currently undergo such surgery^52^. A research survey in the United States showed that only 19.2% of responders supported insurance coverage of metabolic operations^45^.

### Weight stigma and public health policies

Historical examples of illnesses whose social construction incorporated moral judgments about the role of individuals’ actions in contracting the disease (for example, plague, cholera, syphilis, HIV/AIDS), demonstrate that stigma can interfere with public heath efforts to control epidemics^53^. These examples also highlight the importance of initiatives aimed at combatting stigma and social exclusion (for example, United Nations General Assembly Special Session on HIV/AIDS and 2002–2003 World AIDS Campaign)^54^.

Public health efforts to date have typically neglected stigma as a barrier in efforts to address obesity. By contrast, some public health strategies openly embrace stigmatization of individuals with obesity, based on the assumption that shame will motivate them to change behavior and achieve weight loss through a self-directed diet and increased physical exercise^55^. Both observational and randomized-controlled studies show that these strategies can result in the opposite effect, and may instead induce exercise avoidance, consumption of unhealthy diets, and increased sedentary behaviors^1–3^, leading to poor metabolic health, increased weight gain^56,57^, and reduced quality of life^57^.

Some public health messages and government-supported anti-obesity campaigns also characterize the merits of prevention of obesity as a preferable alternative to treatments for established obesity, such as pharmacotherapy or surgery, which are often considered more expensive. This is a misconception, as it frames prevention and treatment as being mutually exclusive, whereas these approaches should generally be directed toward two distinct populations, with different needs.

### Discrimination in employment

Workplace discrimination against individuals with overweight and obesity is common in high-income countries^58^. Individuals with obesity have reported receiving lower starting salaries, can be ranked as less qualified, and can work longer hours than do thinner employees^59^. Persons with obesity can be perceived to be less suitable for employment and are less likely to be invited for an interview^60^, or, if employed, are perceived to be less successful compared with thinner peers^61^. Women with obesity are the especially unlikely to be hired^62^.

A UK study of 119,669 individuals aged 37−73 years found a strong association between higher BMI and lower socioeconomic status, especially in women^63^. Similarly, a US study reported that overweight women are more likely to work in lower-paying jobs and make less money compared with average-size women and all men^64^.

For the vast majority of individuals with obesity who experience discrimination in recruitment or the workplace, there is generally no protection under current legislations^62^. Although some US states have recently introduced a legislation that protects against height and weight discrimination^65^, the Civil Rights Act of 1964 does not identify weight as a protected characteristic, and only in some instances a condition of very high BMI can meet the definition of disability under a 2008 amendment of the Americans with Disabilities Act legislation^66^. This amendment, however, does not cover individuals who are not disabled, even though they can also be victims of weight discrimination.

Similarly, in 2014 the European Court of Justice ruled that being severely overweight could be considered a disability if this condition disrupts an employee’s ability to work. However, obesity per se is generally not specified as a disabling condition in current EU employment law; hence, most anti-discrimination laws require interpretation of whether a person with obesity has a disability. The UK Equality Act (2010)^67^ specifically prohibits discrimination on the grounds of age, disability, gender reassignment, marriage and civil partnership, pregnancy, maternity, race, ethnicity, religion or belief, sex, and sexual orientation—but not for obesity.

### Weight bias and research

Research into obesity and diabetes is underfunded compared with other diseases, relative to their burden and costs on society. For example, the US National Institutes of Health’s projected budgets for cancer, HIV/AIDS, and digestive diseases are 5–10 times greater than the budget for obesity, despite that the latter affecting substantially more Americans.

Among the 5,623 participants in a recent multi-national research survey (the ASK study), higher weight stigma was associated with lower prioritization of spending on obesity research^44^. There are also several ways in which stigma can hinder support of research and scientific advances. For instance, oversimplified notion that obesity is caused by eating too much and exercising too little, implies that the causes of obesity and its epidemic are well-understood, and not complex. In this context, research designed to elucidate etiologic mechanisms of obesity may not be perceived as a priority. Furthermore, funding could be skewed toward projects that are anticipated to be effective (that is, implementation of behavior and lifestyle interventions), reducing support for investigation of novel methods of prevention and treatment or implementation of available evidence-based therapies (that is, pharmaceutical or surgical approaches).

### Causes and contributors for weight stigma

Evidence from several countries^68–71^ shows that when individuals attribute the causes of obesity primarily to internal, controllable factors or personal choices, they exhibit higher weight bias, whereas acknowledging the complex causes of obesity (including elements such as genetics, biology, and environmental factors) is associated with lower levels of weight bias and less blame. These findings suggest that the prevailing narrative of obesity in news coverage, public health campaigns, and political discourse—centered heavily on notions of personal responsibility^72,73^—can play an important part in the expression of weight stigma and reinforce weight-based stereotypes^74^.

The absence of national laws that prohibit weight discrimination can also contribute to expression of weight stigma, as it communicates a societal message that weight stigma is acceptable and tolerable. However, evidence in North America, Europe, Australia, and Iceland suggests that there might be substantial public support to enact and pass legislation to prohibit weight discrimination^75,76^.

### The gap between scientific evidence and misconceptions in the public narrative

The notion that the causes of overweight and obesity depend on individuals’ faults, such as laziness and gluttony, stems from the assumption that body weight is entirely under volitional control. This assumption and many of its corollaries are now at odds with a definitive body of biological and clinical evidence developed over the last few decades.

#### 1. Body weight = calories in – calories out

This equation is often oversimplified in the public narrative of obesity, and even by HCPs, as if the two variables (calories in and calories out) were dependent only on two factors, amount of food consumed and exercise performed, therefore implying that body weight is completely controllable by voluntary decisions to eat less and exercise more.

However, both variables of the equation depend on factors additional to just eating and exercising. For instance, energy intake depends on the amount of food consumed, but also on the amount of food-derived energy absorbed through the gastrointestinal tract, which in turn is influenced by multiple factors, such as digestive enzymes, bile acids, microbiota, gut hormones, and neural signals, none of which are under voluntary control. Similarly, energy output is not entirely accounted for by physical activity, which only contributes to ~30% of total daily energy expenditure. Metabolic rate accounts for 60–80% of total daily energy expenditure, with the thermic effect of feeding constituting approximately 10%^77^. Thus, even when individuals expend energy via exercise, except for elite athletes the overall contribution to energy expenditure is relatively small^78^.

The existence of a powerful, precise homeostatic system that maintains body weight within a relatively narrow, individualized range is supported by scientific evidence. This regulatory system can counteract voluntary efforts to reduce body weight by activating potent compensatory biologic responses (for example, increased appetite and decreased metabolic rate) that promote weight regain. Clinical evidence shows that a 10% weight loss elicits compensatory changes in energy expenditure^79^, and modifications of appetite signals that increase hunger and reduce satiety. These metabolic and biologic adaptations can persist long-term after losing weight and continue even after partial weight regain^80^.

#### 2. Obesity is primarily caused by voluntary overeating and a sedentary lifestyle

Although this concept might appear to be a straightforward conclusion, given common personal experiences of the fluctuations of body weight during periods of excess energy intake or sedentary lifestyle, the evidence supports a more nuanced situation. For example, in a Canadian study that used accelerometers to measure physical activity, girls with obesity took more steps per day than girls within the normal weight range^81^. Similar findings have been observed for adults^82^. Despite substantially higher levels of physical activity, total daily energy expenditure among hunter-gatherers in Africa’s savannahs today is largely similar to that of adults living in modern European or US cities, where obesity prevalence is high^83^. These findings contrast with conventional views that primarily attribute the cause of obesity to sedentary lifestyles and suggest that compensatory metabolic adaptations maintain total energy expenditure relatively constant among human populations and across various levels of physical activities.

Additional evidence is now also available indicating other possible causes and contributors to obesity, including genetic^84^ and epigenetic factors^85^, foodborne factors^86^, sleep deprivation and circadian dysrhythmia^87^, psychological stress, endocrine disruptors, medications, and intrauterine and intergenerational effects. These factors do not require overeating or physical inactivity to explain excess weight^88–90^. A dominant role of genetic factors in obesity pathogenesis has also been demonstrated in studies comparing the concordance of body weight among fraternal versus identical twins^91^, for example, as well as studies of adults adopted as infants compared with their biological and adoptive parents^77,92^. Hence, overeating and reduced physical activity, when present, might be symptoms rather than the root causes of obesity^93^. Finally, the frequent failure of therapeutic and public-health strategies focused on the recommendation to ‘eat less and move more’ should call into question a causal role of voluntary overeating and sedentary lifestyle as primary causes of obesity.

#### 3. Obesity is a lifestyle choice

Persons with obesity typically recognize obesity as a serious health problem, rather than a conscious choice. More than two thirds of 3,008 individuals with obesity surveyed in the ACTION Study considered obesity to be as or more serious than other health conditions, including high blood pressure, diabetes, and depression^94^. Given the negative effects of obesity on quality of life, the well-known risks of serious complications and reduced life expectancy associated with it, it is a misconception to define obesity as a choice.

#### 4. Obesity is a condition, not a disease

Labeling obesity as a disease, risk factor, or condition has implications for treatment and policy development and can contribute to promoting or mitigating stigmatizing views toward affected individuals. An argument often used against labeling obesity a disease is that doing so communicates a societal message that individual responsibility is not relevant in obesity, thus reducing adherence to healthier lifestyles. Defining obesity as a disease, or not, however, should be based on objective medical and biological evidence, not sociologic implications.

The criteria generally used for recognition of disease status are clearly fulfilled in many individuals with obesity as commonly defined, albeit not all. These criteria include specific signs or symptoms (such as increased adiposity), reduced quality of life, and/or increased risk of further illness, complications, and deviation from normal physiology—or well-characterized pathophysiology (for example, inflammation, insulin resistance, and alterations of hormonal signals regulating satiety and appetite).

As reviewed in a statement from the World Obesity Federation^95^, many medical societies as well as the World Health Organization, the US Food and Drug Association, the US National Institutes of Health, and the Nagoya Declaration have now defined obesity as a disease or disease process.

Admittedly, however, defining obesity as a disease, but measuring it only by BMI thresholds (as in contemporary medical practice), risks labeling as ill some individuals who, despite possibly being at risk of future illness, have no current evidence of disease—for example, in cases where high BMI results from being particularly muscular or having short stature. This potential risk of misdiagnosis underscores the inadequacy of current diagnostic criteria for obesity, and the need to identify more meaningful clinical and biological criteria than just BMI to diagnose the disease.

#### 5. Severe obesity is usually reversible by voluntarily eating less and exercising more

This assumption is also not supported by evidence. First, body weight and fat mass are known to be regulated by numerous physiological mechanisms, beyond voluntary food intake and physical exercise. A large body of clinical evidence has shown that voluntary attempts to eat less and exercise more render only modest effects on body weight in most individuals with severe obesity^96,97^. When fat mass decreases, the body responds with reduced resting energy expenditure^79,80^ and changes in signals that increase hunger and reduce satiety^93^ (for example, leptin, ghrelin)^98^. These compensatory metabolic and biologic adaptations promote weight regain and persist for as long as persons are in the reduced-energy state, even if they gain some weight back^98^.

Metabolic surgery is often referred to as an easy way out, based on assumptions that these interventions mechanically restrict food intake in a manner that individuals are not sufficiently disciplined to achieve on their own. However, evidence demonstrates that surgical interventions elicit numerous metabolic effects opposite to the compensatory physiologic responses normally triggered by diet-induced weight reduction, thereby promoting major, long-term weight loss^99^. Such mechanisms include a paradoxical decrease in appetite and increase in metabolic rate, which change adaptively in the opposite directions to those following most non-surgical weight loss^77^. There are also favorable post-operative alterations in gastrointestinal hormones, bile-acid signaling, gut microbiota, absorption and utilization of glucose by the gut, modulations of gastrointestinal nutrient signaling that influence insulin sensitivity, and others^100^.

## Discussion

In this initiative, we sought to inform HCPs, policymakers, and the public about the prevalence, causes, and harmful consequences of weight stigma. A novel, specific goal not formulated in prior related initiatives was to address the gap between popular, stigmatizing narratives around obesity and current scientific knowledge regarding mechanisms of body-weight regulation. We found ample evidence of pervasive weight bias and stigma in many diverse domains of society, causing serious mental and physical harm to individuals with obesity. We met our primary objective of gathering a broad group of experts and scientific organizations to appraise the problem and, to our knowledge for the first time, speak with one voice against this important issue, pledging to do what we can to end it (pledge in Box 1, executive summary in Box 3, and recommendations in Table 3).

There are several limitations to our work. For example, largely owing to the nature of relevant publications, we did not perform a formal systematic review with stringent criteria for levels of evidence. Our method of literature study was closer to a structured rapid review, performed over approximately 6 months, and it only included English-language papers. Also, although our expert panel comprised representatives from ten nations spanning five continents, it was heavily weighted toward individuals from the United States and other high-income countries. Much of the evidence base is also derived from these regions. It is important to note, however, that our final report has been formally endorsed by over 100 organizations at the time of publication (Box 2), including some from low-income and middle-income countries—attesting to the global relevance of the problem and our statements. A strength of our work is that we engaged a diverse group of panelists including academics from disparate disciplines, representatives of patient-advocacy organizations and patients. The broad endorsement of this statement and pledge by a diverse group of organizations, including scientific societies, patient-advocacy groups, academic and medical centers, scientific journals, and a parliamentary group provides an unprecedented opportunity for a concerted effort of all stakeholders to effectively tackle this important problem for medicine and society.

## Conclusions

Weight stigma and discrimination are pervasive and cause significant harm to affected individuals. The widespread narrative of obesity in the media, in public health campaigns, in political discourse, and even in the scientific literature attributing the cause of obesity primarily to personal responsibility has an important role in the expression of societal weight stigma, and reinforces weight-based stereotypes. Weight stigma can mislead clinical decisions, and public health messages, and could promote unproductive allocation of limited research resources. Weight bias and stigma can result in discrimination, and undermine human rights, social rights, and the health of afflicted individuals. Explaining the gap between scientific evidence, and a conventional narrative of obesity built around unproven assumptions and misconceptions might help to reduce weight bias, and its harmful effects. A concerted effort of all stakeholders is required to promote educational, regulatory, and legal initiatives designed to prevent weight stigma and discrimination.

## References

[1] Bauer C, Rosemeier A (2004). A handicap for life - overweight and obesity in pre-school children in Karlsruhe. Gesundheitswesen.

[2] Hayden-Wade HA (2005). Prevalence, characteristics, and correlates of teasing experiences among overweight children vs. non-overweight peers. Obes. Res..

[3] Schvey NA, Puhl RM, Brownell KD (2011). The impact of weight stigma on caloric consumption. Obesity.

[4] Ata RN, Thompson JK (2010). Weight bias in the media: a review of recent research. Obes. Facts.

[5] Gordon TJ (1994). The Delphi method. Futur. Res. Methodol..

[6] Corrigan PW, Watson AC, Barr L (2006). The self-stigma of mental illness: Implications for self-esteem and self-efficacy. J. Soc. Clin. Psychol..

[7] Brewis A, SturtzSreetharan C, Wutich A (2018). Obesity stigma as a globalizing health challenge. Global. Health.

[8] Flint SW, Hudson J, Lavallee D (2015). UK adults’ implicit and explicit attitudes towards obesity: a cross-sectional study. BMC Obes..

[9] Pearl RL (2018). Weight bias and stigma: public health implications and structural solutions. Soc. Issues Policy Rev..

[10] Lydecker JA, O’Brien E, Grilo CM (2018). Parents have both implicit and explicit biases against children with obesity. J. Behav. Med..

[11] Puhl RM, Andreyeva T, Brownell KD (2008). Perceptions of weight discrimination: prevalence and comparison to race and gender discrimination in America. Int. J. Obes..

[12] Spahlholz J, Baer N, König HH, Riedel-Heller SG, Luck-Sikorski C (2016). Obesity and discrimination—a systematic review and meta-analysis of observational studies. Obes. Rev..

[13] Andreyeva T, Puhl RM, Brownell KD (2008). Changes in perceived weight discrimination among Americans, 1995-1996 through 2004-2006. Obesity.

[14] Puhl RM, Himmelstein MS, Quinn DM (2018). Internalizing weight stigma: prevalence and sociodemographic considerations in US adults. Obesity.

[15] Heuer CA, McClure KJ, Puhl RM (2011). Obesity stigma in online news: a visual content analysis. J. Health Commun..

[16] Puhl RM, Luedicke J, Heuer CA (2013). The stigmatizing effect of visual media portrayals of obese persons on public attitudes: does race or gender matter?. J. Health Commun..

[17] Phelan SM (2015). Impact of weight bias and stigma on quality of care and outcomes for patients with obesity. Obes. Rev..

[18] Sabin JA, Marini M, Nosek BA (2012). Implicit and explicit anti-fat bias among a large sample of medical doctors by BMI, race/ethnicity and gender. PLoS One.

[19] Pont SJ, Puhl R, Cook SR, Slusser W (2017). Stigma experienced by children and adolescents with obesity. Pediatrics.

[20] Mannan M, Mamun A, Doi S, Clavarino A (2016). Prospective associations between depression and obesity for adolescent males and females—a systematic review and meta-analysis of longitudinal studies. PLoS One.

[21] Puhl RM, King KM (2013). Weight discrimination and bullying. Best Pract. Res. Clin. Endocrinol. Metab..

[22] Waasdorp TE, Mehari K, Bradshaw CP (2018). Obese and overweight youth: risk for experiencing bullying victimization and internalizing symptoms. Am. J. Orthopsychiatry.

[23] Takizawa R, Danese A, Maughan B, Arseneault L (2015). Bullying victimization in childhood predicts inflammation and obesity at mid-life: a five-decade birth cohort study. Psychol. Med..

[24] Wu YK, Berry DC (2018). Impact of weight stigma on physiological and psychological health outcomes for overweight and obese adults: A systematic review. J. Adv. Nurs..

[25] Papadopoulos S, Brennan L (2015). Correlates of weight stigma in adults with overweight and obesity: a systematic literature review. Obesity.

[26] Jackson SE, Steptoe A, Beeken RJ, Croker H, Wardle J (2015). Perceived weight discrimination in England: a population-based study of adults aged >50 years. Int. J. Obes..

[27] Vartanian LR, Porter AM (2016). Weight stigma and eating behavior: a review of the literature. Appetite.

[28] Major B, Hunger JM, Bunyan DP, Miller CT (2014). The ironic effects of weight stigma. J. Exp. Soc. Psychol..

[29] Han S, Agostini G, Brewis AA, Wutich A (2018). Avoiding exercise mediates the effects of internalized and experienced weight stigma on physical activity in the years following bariatric surgery. BMC Obes..

[30] Sattler KM, Deane FP, Tapsell L, Kelly PJ (2018). Gender differences in the relationship of weight-based stigmatisation with motivation to exercise and physical activity in overweight individuals. Health Psychol. Open.

[31] Jackson SE, Steptoe A (2017). Association between perceived weight discrimination and physical activity: a population-based study among English middle-aged and older adults. BMJ Open.

[32] Sutin AR, Terracciano A (2013). Perceived weight discrimination and obesity. PLoS One.

[33] Hunger JM, Tomiyama AJ (2014). Weight labeling and obesity: a longitudinal study of girls aged 10 to 19 years. JAMA Pediatr..

[34] Quick V, Wall M, Larson N, Haines J, Neumark-Sztainer D (2013). Personal, behavioral and socio-environmental predictors of overweight incidence in young adults: 10-yr longitudinal findings. Int. J. Behav. Nutr. Phys. Act..

[35] Puhl RM (2017). Experiences of weight teasing in adolescence and weight-related outcomes in adulthood: A 15-year longitudinal study. Prev. Med..

[36] Sutin AR, Stephan Y, Luchetti M, Terracciano A (2014). Perceived weight discrimination and C-reactive protein. Obesity.

[37] Jackson SE, Kirschbaum C, Steptoe A (2016). Perceived weight discrimination and chronic biochemical stress: a population-based study using cortisol in scalp hair. Obesity.

[38] Vadiveloo M, Mattei J (2017). Perceived weight discrimination and 10-year risk of allostatic load among US adults. Ann. Behav. Med..

[39] Sutin AR, Stephan Y, Terracciano A (2015). Weight discrimination and risk of mortality. Psychol. Sci..

[40] Gudzune KA, Bennett WL, Cooper LA, Bleich SN (2014). Patients who feel judged about their weight have lower trust in their primary care providers. Patient Educ. Couns..

[41] Puhl R, Peterson JL, Luedicke J (2013). Motivating or stigmatizing? Public perceptions of weight-related language used by health providers. Int. J. Obes..

[42] Aldrich T, Hackley B (2010). The impact of obesity on gynecologic cancer screening: an integrative literature review. J. Midwifery Womens Health.

[43] lberga A, Edache I, Forhan M, Russell-Mayhew S (2019). Weight bias and health care utilization: A scoping review. Prim. Health Care Res. Dev..

[44] O’Keeffe, M., Flint, S.W., Watts, K. & Rubino, F. Knowledge gaps and weight stigma shape attitudes toward obesity: insights from the ASK study. *Lancet Diabetes Endocrinol.*10.1016/S2213-8587(20)30073-5 (2020). 10.1016/S2213-8587(20)30073-5 32142624

[45] Dolan P (2019). Assessment of public attitudes toward weight loss surgery in the United States. JAMA Surg..

[46] Kim JJ (2017). Evidence base for optimal preoperative preparation for bariatric surgery: does mandatory weight loss make a difference?. Curr. Obes. Rep..

[47] Keith CJ, Goss LE, Blackledge CD, Stahl RD, Grams J (2018). Insurance-mandated preoperative diet and outcomes after bariatric surgery. Surg. Obes. Relat. Dis..

[48] Vartanian LR, Fardouly J (2013). The stigma of obesity surgery: negative evaluations based on weight loss history. Obes. Surg..

[49] Hansen BB, Dye MH (2018). Damned if you do, damned if you don’t: the stigma of weight loss surgery. Deviant Behav..

[50] Rubino F (2016). Metabolic surgery in the treatment algorithm for type 2 diabetes: a joint statement by international diabetes organizations. Diabetes Care.

[51] Sjostrom L (2013). Review of the key results from the Swedish Obese Subjects (SOS) trial: a prospective controlled intervention study of bariatric surgery. J. Intern. Med..

[52] Dixon JB (2016). Regional differences in the coverage and uptake of bariatric-metabolic surgery: A focus on type 2 diabetes. Surg. Obes. Relat. Dis..

[53] Gruskin S, Mann J, Tarantola D (1998). Past, present, and future: AIDS and human rights. Health Hum. Rights.

[54] Parker R, Aggleton P (2003). HIV and AIDS-related stigma and discrimination: a conceptual framework and implications for action. Soc. Sci. Med..

[55] Callahan D (2013). Obesity: chasing an elusive epidemic. Hastings Cent. Rep..

[56] Puhl RM, Heuer CA (2009). The stigma of obesity: a review and update. Obesity.

[57] Puhl R, Suh Y (2015). Health consequences of weight stigma: implications for obesity prevention and treatment. Curr. Obes. Rep..

[58] Rudolph CW, Wells CL, Weller MD, Baltes BB (2009). A meta‐analysis of empirical studies of weight‐based bias in the workplace. J. Vocat. Behav..

[59] Schulte PA (2007). Work, obesity, and occupational safety and health. Am. J. Public Health.

[60] Agerström J, Rooth DO (2011). The role of automatic obesity stereotypes in real hiring discrimination. J. Appl. Psychol..

[61] Flint SW, Snook J (2014). Obesity and discrimination: the next ‘big issue’?. International J. Discrimination Law.

[62] Flint SW (2016). Obesity discrimination in the recruitment process: “You’re not hired!”. Front. Psychol..

[63] Tyrrell J (2016). Height, body mass index, and socioeconomic status: mendelian randomisation study in UK Biobank. Br. Med. J..

[64] Shinall, J. B. Occupational characteristics and the obesity wage penalty. Vanderbilt Law and Economics Research Paper No. 16–12; Vanderbilt Public Law Research Paper No. 16–23, https://ssrn.com/abstract=2379575 (7 October 2015).

[65] Rushing, B. et al. An Act making discrimination on the basis of height and weight unlawful. Commonwealth of Massachusetts Bill H.952, https://malegislature.gov/Bills/190/H952 (18 January 2017).

[66] Pomeranz JL, Puhl RM (2013). New developments in the law for obesity discrimination protection. Obesity.

[67] Equality and Human Rights Commission. Equality Act 2010. http://www.legislation.gov.uk/ukpga/2010/15/contents (2010).

[68] Persky S, Eccleston CP (2011). Impact of genetic causal information on medical students’ clinical encounters with an obese virtual patient: health promotion and social stigma. Ann. Behav. Med..

[69] Hilbert A (2009). Causal attributions of obese men and women in genetic testing: implications of genetic/biological attributions. Psychol. Health.

[70] Teachman BA, Gapinski KD, Brownell KD, Rawlins M, Jeyaram S (2003). Demonstrations of implicit anti-fat bias: the impact of providing causal information and evoking empathy. Health Psychol..

[71] Puhl RM (2015). A multinational examination of weight bias: predictors of anti-fat attitudes across four countries. Int. J. Obes..

[72] Barry CL, Jarlenski M, Grob R, Schlesinger M, Gollust SE (2011). News media framing of childhood obesity in the United States from 2000 to 2009. Pediatrics.

[73] Kim SH, Willis LA (2007). Talking about obesity: news framing of who is responsible for causing and fixing the problem. J. Health Commun..

[74] Blaine B, McElroy J (2002). Selling stereotypes: weight loss infomercials, sexism, and weightism. Sex Roles.

[75] Puhl RM (2015). Potential policies and laws to prohibit weight discrimination: public views from 4 countries. Milbank Q..

[76] Hilbert A (2017). Public support for weight-related antidiscrimination laws and policies. Obes. Facts.

[77] Morton GJ, Cummings DE, Baskin DG, Barsh GS, Schwartz MW (2006). Central nervous system control of food intake and body weight. Nature.

[78] Hall KD (2012). Energy balance and its components: implications for body weight regulation. Am. J. Clin. Nutr..

[79] Leibel RL, Rosenbaum M, Hirsch J (1995). Changes in energy expenditure resulting from altered body weight. N. Engl. J. Med..

[80] Hall KD, Kerns JC, Brychta R, Knuth ND (2016). Response to “Overstated metabolic adaptation after ‘The Biggest Loser’ intervention”. Obesity.

[81] Colley RC (2011). Physical activity of Canadian children and youth: accelerometer results from the 2007 to 2009 Canadian Health Measures Survey. Health Rep..

[82] Colley RC (2011). Physical activity of Canadian adults: accelerometer results from the 2007 to 2009 Canadian Health Measures Survey. Health Rep..

[83] Pontzer H (2012). Hunter-gatherer energetics and human obesity. PLoS One.

[84] Maes HH, Neale MC, Eaves LJ (1997). Genetic and environmental factors in relative body weight and human adiposity. Behav. Genet..

[85] Heindel JJ, Newbold R, Schug TT (2015). Endocrine disruptors and obesity. Nat. Rev. Endocrinol..

[86] Chassaing B (2015). Dietary emulsifiers impact the mouse gut microbiota promoting colitis and metabolic syndrome. Nature.

[87] Arble DM, Bass J, Laposky AD, Vitaterna MH, Turek FW (2009). Circadian timing of food intake contributes to weight gain. Obesity.

[88] Tremblay A, Chaput JP (2008). About unsuspected potential determinants of obesity. Appl. Physiol. Nutr. Metab..

[89] Keith SW (2006). Putative contributors to the secular increase in obesity: exploring the roads less traveled. Int. J. Obes..

[90] McAllister EJ (2009). Ten putative contributors to the obesity epidemic. Crit. Rev. Food Sci. Nutr..

[91] Börjeson M (1976). The aetiology of obesity in children. A study of 101 twin pairs. Acta Paediatr. Scand..

[92] Stunkard AJ (1986). An adoption study of human obesity. N. Engl. J. Med..

[93] Sharma AM, Padwal R (2010). Obesity is a sign - over-eating is a symptom: an aetiological framework for the assessment and management of obesity. Obes. Rev..

[94] Kaplan LM (2018). Perceptions of barriers to effective obesity care: results from the national ACTION study. Obesity.

[95] Bray GA, Kim KK, Wilding JPH, World Obesity, F. (2017). Obesity: a chronic relapsing progressive disease process. A position statement of the World Obesity Federation. Obes. Rev..

[96] Franz MJ (2007). Weight-loss outcomes: a systematic review and meta-analysis of weight-loss clinical trials with a minimum 1-year follow-up. J. Am. Diet. Assoc..

[97] Wadden TA (2011). A two-year randomized trial of obesity treatment in primary care practice. N. Engl. J. Med..

[98] Sumithran P (2011). Long-term persistence of hormonal adaptations to weight loss. N. Engl. J. Med..

[99] Cummings DE, Rubino F (2018). Metabolic surgery for the treatment of type 2 diabetes in obese individuals. Diabetologia.

[100] Batterham RL, Cummings DE (2016). Mechanisms of Diabetes Improvement Following Bariatric/Metabolic Surgery. Diabetes Care.

